# Identification of novel mutation in *RANKL* by whole‐exome sequencing in a Thai family with osteopetrosis; a case report and review of *RANKL* osteopetrosis

**DOI:** 10.1002/mgg3.1727

**Published:** 2021-05-30

**Authors:** Pongtawat Lertwilaiwittaya, Bhoom Suktitipat, Phongphak Khongthon, Warut Pongsapich, Chanin Limwongse, Manop Pithukpakorn

**Affiliations:** ^1^ Department of Medicine Faculty of Medicine Siriraj Hospital Mahidol University Bangkok Thailand; ^2^ Department of Biochemistry Faculty of Medicine Siriraj Hospital Mahidol University Bangkok Thailand; ^3^ Integrative Computational BioScience Center Mahidol University Bangkok Thailand; ^4^ Research Division Faculty of Medicine Siriraj Hospital Mahidol University Bangkok Thailand; ^5^ Department of Otorhinolaryngology Faculty of Medicine Siriraj Hospital Mahidol University Bangkok Thailand; ^6^ Siriraj Center of Research Excellence in Precision Medicine Faculty of Medicine Siriraj Hospital Mahidol University Bangkok Thailand

**Keywords:** bone, genome, osteopetrosis, skeletal dysplasia, *TNFSF11*

## Abstract

**Background:**

Osteopetrosis is a rare form of skeletal dysplasia characterized by increased bone density that leads to bone marrow failure, compressive neuropathy, and skeletal dysmorphism. Molecular diagnosis is essential as it guides treatment and prognosis. We report Thai siblings with an ultra‐rare form of osteopetrosis.

**Methods:**

The older brother and the younger sister presented with chronic mandibular osteomyelitis in their 20s. Since childhood, they had visual impairment, pathological fracture, and skeletal dysmorphism.

Quadruplet whole‐exome sequencing was performed and confirmed with Sanger sequencing. Novel mutation in *TNFSF11* (*RANKL*) c.842T>G, p.Phe281Cys was identified in a homozygous state in both siblings.

**Results:**

Surgical debridement, antibiotic, and hyperbaric oxygen therapy were used and discontinued over a 6‐month period with normalization of C‐reactive protein. Hematopoietic stem cell transplantation candidacy was excluded by molecular diagnosis.

**Conclusion:**

We report a novel mutation in an ultra‐rare form of osteopetrosis. Our siblings manifested with a milder phenotype in comparison with nine cases previously published.

## Graphical abstract

A novel mutation in an ultra‐rare form of RANKL osteopetrosis with a mild phenotype in a Thai family was reported. The spectrum of RANKL osteopetrosis, including all nine previously published cases, was reviewed.

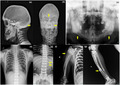

**Abbreviations**
**P1NP: procollagen‐1N‐terminal peptide
ACMG: American College of Medical Genetics and Genomics
AP: anteroposterior
ARO: autosomal recessive osteopetrosis
BUN: blood urea nitrogen
CT: computerized tomography
dB: decibel
Hb: hemoglobin concentration
PTH: parathyroid hormone
*RANKL*
: (gene) receptor activator of nuclear factor kappa‐B ligand
SNVs: single‐nucleotide variants
*TNFSN11*
: (gene) tumor necrosis factor ligand superfamily member 11

## 1 INTRODUCTION

Osteopetrosis (OMIM number #259710) is a rare form of skeletal dysplasia and is characterized by impairment of osteoclastogenesis or dysfunction of osteoclastic bone resorption activity. This results in abnormally high bone mineral density which leads to bone brittleness to tensile force, bone marrow failure from decreased marrow space, and compressive neuropathy especially optic atrophy. Osteopetrosis is classified clinically into three forms: malignant autosomal recessive infantile, benign autosomal dominant adult‐onset, and intermediate. Currently, diagnosis is guided by recommendations from the osteopetrosis working group which include radiographic features from skeletal survey and molecular diagnosis (Wu et al., 2017). The genetic basis of osteopetrosis was previously reported from multiple studies in osteopetrosis cohort, and the disease‐causing genes were classified into osteoclast‐rich group (*TCIRG1*, *CLCN7*, *OSTM1*, *SNX10*, *PLEKHM1*, *FERMT3*, *LRRK1*, *MITF*, *C16orf57*, *CAII*, *RELA*, *CTSK*, *NEMO*, and *IKBKG*) and osteoclast‐poor group (*RANKL*, *RANK*, *SLC29A3*, *TRAF6*, and *CSF1R*; Penna et al., 2019; Sobacchi et al., 2013). Genetic testing in osteopetrosis is recommended as it helps to confirm the diagnosis and delineate the clinical spectrum, providing information about major organ involvement, benefit of hematopoietic stem cell transplantation, and reproductive risk (Wu et al., 2017). Here we report two siblings with RANKL‐dependent osteopetrosis and review the phenotypic spectrum of this rare form of osteopetrosis.

## 2 MATERIAL AND METHOD

### 2.1 Sample collection and DNA extraction

Sample collection was performed on 28 December 2017.

### 2.2 Whole‐exome sequencing and bioinformatics analysis

Whole‐exome sequencing and bioinformatics were carried out on 22 June 2018 to 25 December 2018.

Sanger sequencing report was obtained on 5 September 2018.

Re‐analysis of whole‐exome sequencing was carried out on March 10 2021.

## 3 RESULT

### 3.1 Case presentation

Two siblings of older brother and younger sister were referred to Siriraj Hospital for evaluation of their multifocal recurrent chronic osteomyelitis of the mandible. The patients were admitted to the otorhinolaryngology ward for surgical management. Upon admission, the attending physician found both patients to have gross skeletal dysmorphism and visual impairment. Medical genetics consultation was requested to evaluate possible causes of skeletal dysplasia in these siblings.

#### 3.1.1 Case 1: The older brother

The 26‐year‐old Thai male was born to a family without a history of hereditary disease or short stature. He developed multiple draining sinuses from his oral cavity and lower jaw since age 18. The patient had been repeatedly treated with oral antibiotics and local wound care at his community hospital for 4 years. He had painless progressive bilateral visual loss since age 9. Upon unsuccessful treatment of infection, he was referred to the state hospital where he was found to have pathological fracture of both tibias after a minor injury. Skeletal X‐ray also showed abnormal bone density. He was finally referred to our hospital after an unsuccessful 3‐year course of intravenous antibiotics and multiple surgical procedures of his jaw. Upon arrival, the patient was found to have an enlarged mandible with multiple cutaneous submandibular fistulas, palpable condyles, and exposed necrotic bone at the lower alveolar ridge. His visual impairment had progressed to retaining only light perception in both eyes.

**FIGURE 1:**
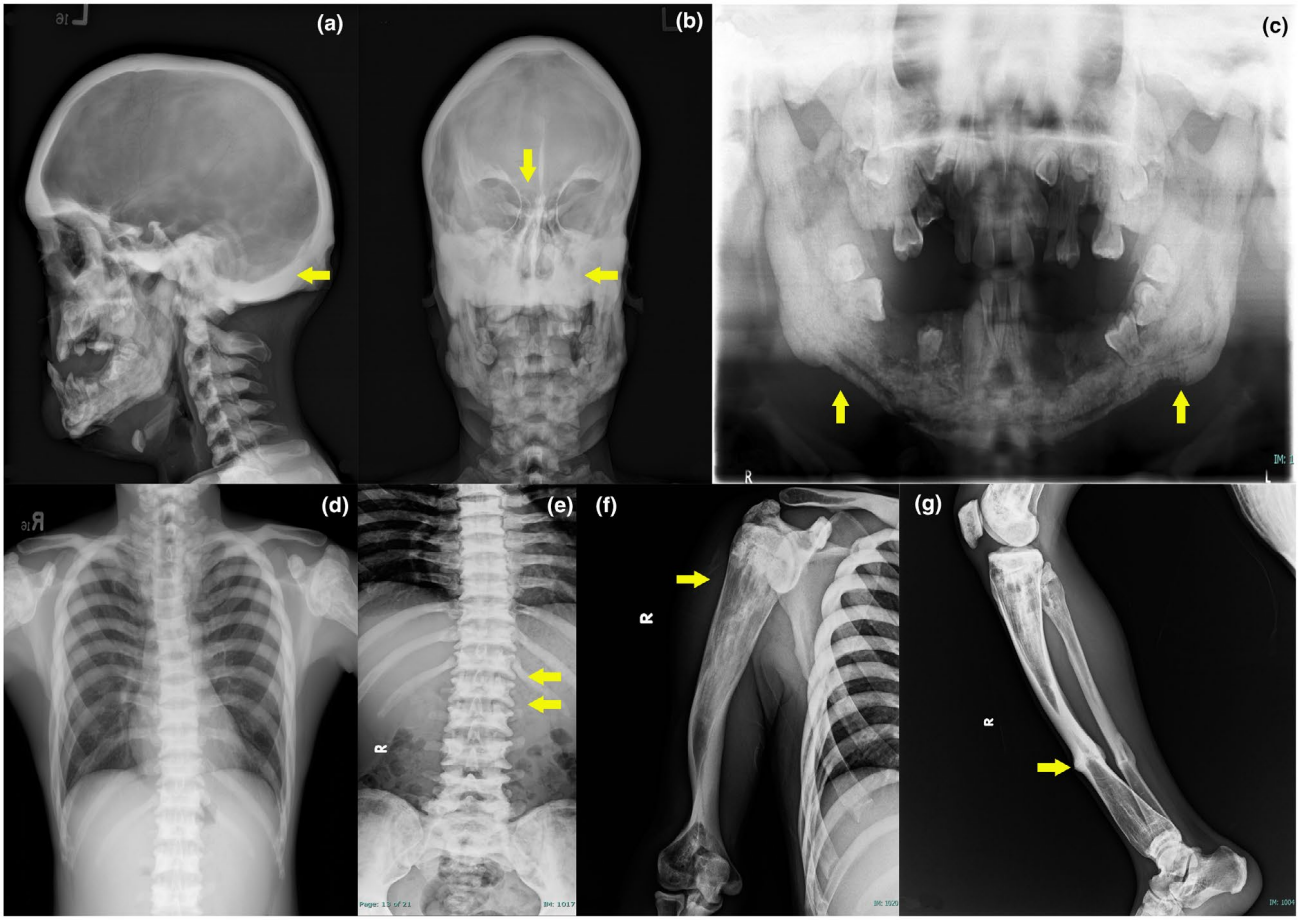
Skeletal survey of the older brother. (a) X‐ray AP view of skull (arrow: thickening bony calvarium). (b) X‐ray Lateral view of skull (arrows: hypoplasia of paranasal air sinuses). (c) X‐ray Panoramic view of mandible (arrows: cortical disruption of mandible and missing teeth from osteomyelitis). (d) X‐ray of chest. (e) X‐ray AP view of TL spine (arrows: sandwich vertebrae). (f) X‐ray of upper extremities (arrow: Erlenmeyer flask deformity; metaphyseal flaring of right Humerus). (g) X‐ray of lower extremities (arrow: healed pathological fracture of right Tibia)

Physical inspection of the patient demonstrated a man with short stature (130 cm height; less than 3rd percentile) with facial dysmorphism including bitemporal narrowing, bilateral exotropia, and exophthalmos. Hypermobility of proximal interphalangeal joints of both hands and bilateral bow legs were noted. Ophthalmological examination revealed exophthalmos of 27 mm for right eye and 29 mm for left eye measured with Hertel exophthalmometer. Limited extraocular movement of both eyes especially on upward gaze (5–10 degrees) was observed. Bilateral pale optic disc consistent with optic atrophy was noted on dilated fundoscopic examination. Otorhinolaryngological examination revealed lateralization to the right from Weber test, superior bone conduction versus air conduction of sound from Rinne test on both sides, pure tone audiometry masking level of 40 dB on the right ear and 32 dB on the left ear, and 100% unaided recognition score speech audiometry in both ears which overall suggested a presence of bilateral conductive hearing loss. X‐ray imaging of the skull in AP and lateral view, mandible panoramic view, and extremity bone survey demonstrated dense sclerotic change of all bones (illustrated in Figure 1). Further CT scan of the head and neck revealed diffuse cortical thickening and dense sclerotic change of bony calvarium, visualized facial bones, cervicothoracic spine and bony thoraxes, hypoplasia of maxillary bone and maxillary sinus, and severe stenosis of skull base canal including internal auditory meatus, bilateral orbital apices and optic canals. Initial laboratory investigation revealed mostly normal results: complete blood count was Hb 13 g/dl (12.7–16.9 g/dl), white blood cell count 8,000 cells/μl (4,500–11,300 cells/μl) and platelet count 189,000/μl (160,000–356,000/μl), BUN 6.1 mg/dl (6–20 mg/dl), serum creatinine 0.52 mg/dl (0.67–1.17 mg/dl), total calcium 9.1 mg/dl (8.6–10.0 mg/dl), phosphorus 2.8 mg/dl (2.5–4.5 mg/dl) and high PTH level at 82.7 pg/ml (15.0–65.0 pg/ml). Serum electrolyte, liver function test, thyroid function test, and urinalysis were in normal range. Bone turnover biomarkers were osteocalcin (N‐mid) 17.90 ng/ml (24–70 ng/ml), beta‐crosslaps 0.059 ng/ml (0.016–0.0548 for male age 30–50 year), and **P1NP 20.90 ng/ml.

Over the next 6 months, the patient underwent five surgical debridements with tooth extraction and 40 courses of hyperbaric chamber treatment. Tissue samples (mandible, tooth, and skin) were sent for pathological examination which revealed chronic osteomyelitis. Aerobic and anaerobic mycobacterial and fungal cultures revealed oral microbiota (*Proteus mirabilis*, *Citrobacter koseri*, *Enterococcus* *faecalis*, *Bifidobacterium* spp., *Parvimonas vicar*, and later methicillin‐resistant *Staphylococcus aureus*). Ciprofloxacin 400 mg every 12 hr and Clindamycin 600 mg three times daily were prescribed and discontinued after a consecutive normalization of C‐reactive protein over 6‐month period (from 9.88 to 1.29 to 1.09 mg/L). The patient remained antibiotic free for a year up to the current follow up. His visual impairment has been clinically stable with an active follow‐up plan annually with ophthalmologist. His lymphocyte subpopulation test after a year free of antibiotic was CD4^+^ Cell 23.46% (24.1%–50.70%), Absolute CD4^+^ T cell count 712 cells/μl (470–1,404 cells/μl), CD8^+^ Cell 34.15% (17.10%–44.60%), Absolute CD8^+^ T cell count 1,036 cells/μl (360–1,250 cells/μl), CD4^+^/CD8^+^ ratio 0.69 (0.65–2.49), CD3^+^ Cell 58.84% (46.20%–82.70%), Absolute CD3^+^ T cell count 1,786 cells/μl (960–2,430 cells/μl), CD19^+^ 27.01% (7.70%–25.40%), and Absolute B cell count 820 cells/μl (140–660 cells/μl).

#### 3.1.2 Case 2: The younger sister

A 19‐year‐old Thai female who is the younger sister of Case 1 was evaluated. She had a similar course of disease with earlier onset. The patient was noted to have fractures of both shafts of tibia at age 15 without history of prior trauma. She also developed multiple draining sinuses from her oral cavity and lower jaw at that time. The patient was similarly treated with oral antibiotics and local wound care for 4 years, later referred to the state hospital, and lastly referred to our hospital. She also had progressive painless bilateral visual impairment since age 12.

**FIGURE 2:**
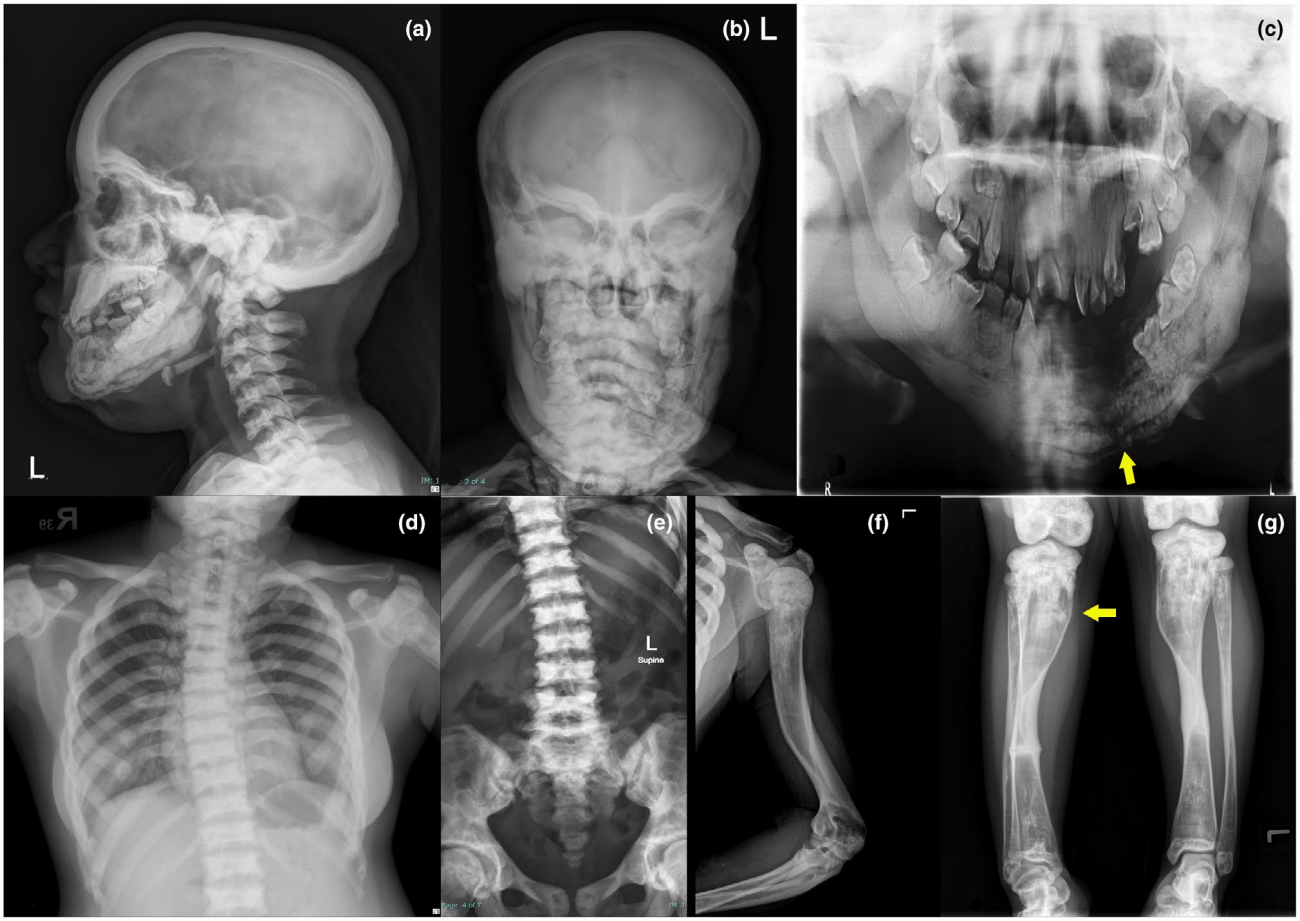
Skeletal survey of the younger sister. (a) X‐ray AP view of skull. (b) X‐ray Lateral view of skull. (c) X‐ray Panoramic view of mandible (arrow: cortical disruption of mandible and missing teeth from osteomyelitis). (d) X‐ray of chest. (e) X‐ray AP view of TL spine. (f) X‐ray of upper extremities. (g) X‐ray of lower extremities (Both Tibia) (arrow: metaphyseal flaring of right Tibia)

Physical examination revealed a female with short stature (128 cm height; less than 3rd percentile) with facial dysmorphism including bitemporal narrowing and exophthalmos. Bilateral bow legs were also noted. Ophthalmological examination revealed bilateral exophthalmos with bilateral optic atrophy. Otorhinolaryngological examination revealed no lateralization of Weber test, air conduction was better than bone conduction from Rinne test both sides, right ear 17 dB and left ear 17 dB pure tone audiometry masking level, both 100% unaided recognition score speech audiometry which all suggested for an absence of hearing loss. X‐ray imaging of skull AP and lateral view, mandible panoramic view, and extremity bone survey were performed and found dense sclerotic changes (illustrated in Figure 2). Further CT scan of the head and neck revealed diffuse cortical thickening and dense sclerotic changes of bony calvarium, mixed osteolytic, and osteosclerotic lesion, surrounding with thickened solid periosteal reaction at body of mandible extending to both rami (left was more severe than right) and head of left mandible, and associated non‐enhancing soft tissue thickening along the mixed osteolytic and osteosclerotic lesion. These mandibular lesions were suggestive of chronic osteomyelitis. Initial laboratory investigation was in normal range; complete blood count Hb 12.6 g/dl (12.7–16.9 g/dl), white blood cell count 9,040/μl (4,500–11,300 cells/μl) and platelet count 369,000/μl (160,000–356,000/μl), BUN 9.3 mg/dl (6–20 mg/dl), serum creatinine 0.44 mg/dl (0.67–1.17 mg/dl), total calcium 8.6 mg/dl (8.6–10.0 mg/dl), phosphorus 3.5 mg/dl (2.5–4.5 mg/dl) and normal PTH level of 44.57 pg/ml (15.0–65.0 pg/ml). Serum electrolyte, liver function test, thyroid function test, and urinalysis were in normal range. Bone turnover biomarkers were also within range: osteocalcin (N‐mid) 14.70 ng/ml (11–43 ng/ml), beta‐crosslaps 0.037 ng/ml (0.025–0.573 ng/ml), and **P1NP 18.60 ng/ml (15.13–58.59 ng/ml).

Over the next 6 months, the patient underwent multiple surgical debridement with tooth extraction and 40 courses of hyperbaric chamber treatment. The tissue from mandible curettage was sent for pathological examination which revealed necrotic bone and inflammatory tissue, consistent with chronic osteomyelitis. Aerobic and anaerobic mycobacterial and fungal cultures revealed oral microbiota (*Pseudomonas aeruginosa*, *Hemophilus parainfluenzae*, *Prevotella* spp., and later methicillin‐resistant *S. aureus*). Ciprofloxacin 400 mg every 12 hr and Clindamycin 600 mg three times daily were prescribed and discontinued over a consecutive normalization of C‐reactive protein over a 6‐month period (from 8.72 to <0.6 mg/L). The patient remained antibiotic free for a year up to the current follow‐up. Her visual impairment has been clinically stable and she has also been recommended to follow‐up with an ophthalmologist annually. Her lymphocyte subpopulation test after a year free of antibiotic was CD4+ Cell 21.86% (24.1%–50.70%), absolute CD4+ T cell count 566 cells/μl (470–1,404 cells/μl), CD8+ Cell 38.37% (17.10%–44.60%), absolute CD8+ T cell count 994 cells/μl (360–1,250 cells/μl), CD4+/CD8+ ratio 0.57 (0.65–2.49), CD3+ Cell 63.40% (46.20%–82.70%), absolute CD3+ T cell count 1,643 cells/μl (960–2,430 cells/μl), CD19+ 14.86% (7.70%–25.40%), and absolute B cell count 385 cells/μl (140–660 cells/μl).

**FIGURE 3:**
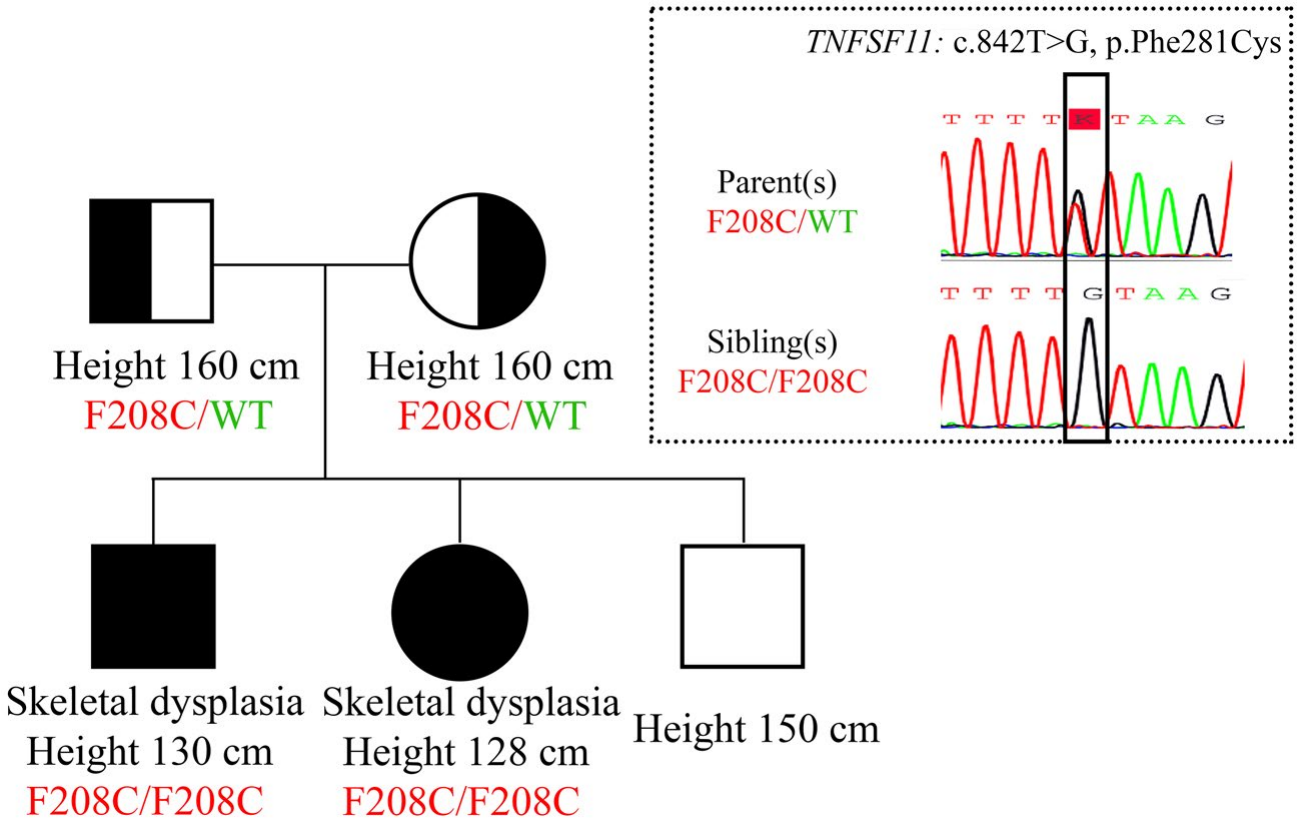
Pedigree demonstrating height and the results of Sanger sequencing of *TNFSF11* (NM_003701.4)

The pedigree of this family with individual height in centimeters is shown in Figure 3. History of consanguinity was not reported in this family. The inheritance pattern was consistent with autosomal recessive inheritance. Abnormal findings were apparently compatible with osteopetrosis spectrum of skeletal dysplasia. Since there are over 400 heritable disorders in skeletal dysplasia spectrum, we chose clinical exome sequencing as a preferred diagnostic tool. We performed a quadruplet whole‐exome sequencing in both affected siblings and both of their parents to determine causative gene of this autosomal recessive skeletal dysplasia.

### 3.2 Whole‐exome sequencing analysis

The genomic DNA of four individuals, including two skeletal dysplasia patients and their parents, was enriched for the exonic region using the Agilent SureSelect Human All Exon V5+UTRs. Captured libraries were sequenced on an Illumina HiSeq 2500/4000. ANNOVAR was adopted to annotate SNVs and indels according to the databases. Subsequently, ~130,000 annotated variants from each individual were filtered to exclude common and known benign and likely benign variants. We utilized VarAFT software (Desvignes et al., 2018) for variant filtration. We used “osteopetrosis” as a Human Phenotype Ontology (HPO) term (HP:0011002) to screen for relevant genes and set the allele frequency threshold to be less than 0.01. Because both parents were unaffected, mode of inheritance was likely autosomal recessive. All filtered variants identified in as homozygous or compound heterozygous in both patients and heterozygous in each parent were chosen to be potential candidates. There was only one variant; *TNFSF11* (*RANKL*; OMIM number *602642) (NM_003701.4): c.842T>G, p.Phe281Cys, left after we applied all aforementioned filters.

*TNFSF11* (tumor necrosis factor ligand superfamily member 11), also known as *RANKL* (Receptor activator of nuclear factor kappa‐β ligand), is known to be a crucial growth factor for osteoclast differentiation and activation (Liu & Zhang, 2015). In addition to its receptor RANK, RANKL could bind to leucine‐rich repeat‐containing G‐protein‐coupled receptor 4 (LGR4) that exerts its action via the GSK3‐β (glycogen synthase kinase 3) signaling pathway which inhibit osteoclast differentiation (Luo et al., 2016). A link between *TNFSF11* and autosomal recessive osteoclast‐poor osteopetrosis was established in 2007 (Sobacchi et al., 2007). The variants NM_003701.4: c.842T>G, p.Phe281Cys (Position 13:42606806) found in this family was novel (absence in gnomAD, 1000Genomes and ClinVar). This variant is also absence in 384 ethnic‐matched chromosomes from Thai cohorts of 25 sudden unexpected death syndrome, 60 disseminated non‐tuberculous mycobacterial disease, and 102 retinitis pigmentosa patients (Jinda et al., 2014; Pithukpakorn et al., 2015; Suktitipat et al., 2017). Multiple computational prediction tools suggested deleteriousness of this variants (CADD score 24.702, MetaSVM 1.056). The amino acid in this position is conserved among vertebrates. This residue falls in the tumor necrosis factor‐like core domain, a signifying domain of RANKL. Specifically, the variants code for amino acid that forms a secondary structure β‐strands F which is important in trimerization of RANKL. There was a study that demonstrates an impairment of osteoclast differentiation and activation when a truncated recombinant RANKL that lacked β‐strands F was used (Cheng et al., 2009). Furthermore, all previous reports of *RANKL* variants in families with RANKL‐dependent osteopetrosis were variants that code for the TNF‐like core domain (Table 1). We further performed Sanger sequencing to confirm the presence of homozygous SNV in *TNFSF11* (NM_003701.4: c.842T>G, p.Phe281Cys) in both patients, and the presence of heterozygous SNV in each parents (Figure 3). The variant was finally classified as likely pathogenic by PM1, PM2, PM3, PP1, PP2, and PP3 criteria (Richards et al., 2015). In conclusion, quadruplet whole‐exome sequencing analyses in this family identified a novel likely pathogenic variant c.842T>G, p.Phe281Cys in *TNFSF11* gene. Patients were diagnosed with RANKL‐dependent osteoclast‐poor autosomal recessive osteopetrosis.

**TABLE 1:** Clinical spectrum of published cases of RANKL‐dependent osteopetrosis

Patient	Ethnic origin	Variants Nomenclature *RANKL* (NM_003701.3)	Age of onset	CNS/Neuropathies	Serum Calcium (mg/dl)	Skeletal dysmorphism	Hematology	Status
S1^a^	Tunisian (Consanguinous)	c.532+4_532+8delAGCT, r.434_532del Exon 7 skipping	2 days to 1 year	Severe cerebro‐bulbar deterioration	ND	ND	Hepatosplenomegaly	11 years old, loss follow‐up (2013)
S2A^a^	Sikh family	c.596T>A, p.Met199Lys		Blind	ND	Severe growth retardation not response to GH, Osteomyelitis of Toe, Severely impaired dentition	Hemolytic anemia, Hepatosplenomegaly	18 years old (2013)
S2B				Blind, Hydrocephalus	ND	Severe growth retardation, Delayed dentition with chronic parotid/mandibular abscess	Anemia, Hepatosplenomegaly, Normal B cell and T cell subset, T cell proliferation and propensity to apoptosis	13 years old (2013)
S3A^a^	Kurdish (Consanguinous)	c.828_829delCG, p.Val277Trpfs*5		Blind	Hypocalcemia	Severe growth retardation, Defective dentition	Bicytopenia, Hepatosplenomegaly	>19 years old (2013)
S3B				ND	ND	Severe growth retardation, Defective dentition	Hepatosplenomegaly	6 years old (2013)
S4	Sikh family	c.596T>A, p.Met199Lys		Blind	ND	Moderate growth retardation (P4th), Delayed tooth eruption	Normal B cell and T cell subset, T cell proliferation and propensity to apoptosis, Hepatosplenomegaly	6.5 years old (2013)
S5 & S6	ND	c.596T>A, p.Met199Lys	ND	ND	ND	ND	ND	ND
S7^a^	Lebanese (Consanguinous)	c.667C>T, p.Arg223*	Soon after birth	Cranial nerve involvement	Hypocalcemic seizure	Severe growth retardation, Defective dentition	ND	22 years old (2013)
Elder brother	Thai	c.842T>G, p.Phe281Cys	Childhood	Optic atrophy	9.1	Short stature, Jaw osteomyelitis	Normal	26 years old (2020)
Younger sister	Thai	c.842T>G, p.Phe281Cys	Childhood	Optic atrophy	8.6	Short stature, Jaw osteomyelitis	Normal	19 years old (2020)

Abbreviation: ND, not demonstrated.

^a^ Patient who received hematopoietic stem cell transplantation.

**FIGURE 4:**
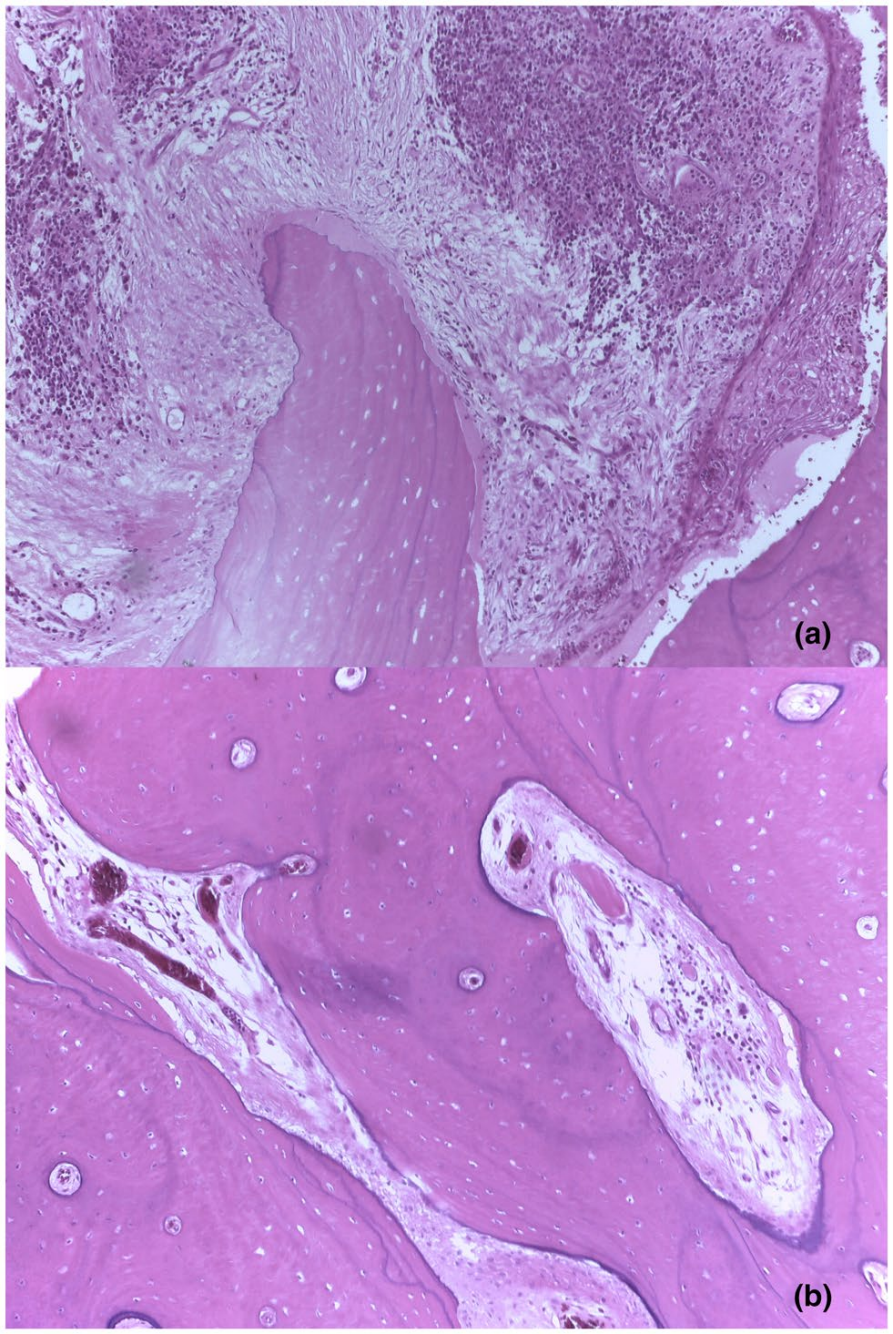
Pathological examination of the elder brother's surgical specimen (H&E stain, 100×). (a) Necrotic bone in the background of chronic non‐specific inflammation; consistent with chronic osteomyelitis. (b) Viable bone with fibrovascular tissue. No osteoclasts observed; consistent with osteoclast‐poor nature of the disease

After the result from Sanger sequencing came out, we further requested a review of pathological specimen from both patients to confirm the osteoclast‐poor nature of this disease. In Figure 4, we demonstrated an H&E stain of surgical tooth extraction specimen from the elder brother. There was an evidence of necrotic bone surrounded by chronic non‐specific inflammation, consistent with chronic mandibular osteomyelitis. As for the remaining viable bone with fibrovascular tissue, there were no osteoclasts observed. The pathological finding in the younger sister is in accordance to the elder brother's. Overall, the pathological examination revealed an absence of osteoclast, which was in consonance with the finding of homozygous likely pathogenic variants in *TNFSF11* gene from the quadruple whole‐exome sequencing.

## 4 DISCUSSION

Overall, chronic recurrent osteomyelitis of mandible has multiple etiologies including inadequate antibiotic treatment, inadequate surgical debridement, retained infective teeth, insufficient vascular supply, and metabolic bone disease. Genetic predisposition was suggested to play an important role in a case report of mandibular osteomyelitis in monozygotic twins (Ylikontiola et al., 1994). There was no consensus whether which case of mandibular osteomyelitis should be referred for genetic evaluation. However, taken with an occurrence in sibling and a presence of skeletal dysmorphism as in our cases, further genetic evaluation should be recommended.

Mandibular osteomyelitis is a significant manifestation of osteopetrosis, but not limited to RANKL‐dependent osteopetrosis (Carvalho et al., 2018; Dunphy et al., 2019; Machado Cde et al., 2015). The pathophysiology is likely similar to an osteonecrosis of jaws associated with bisphosphonates or denosumab (RANKL inhibitor), in which the mechanism of action is inhibition of osteoclast function. Continuous exposure to oral flora with thin connective tissue protection between periosteum and oral mucosa could explain for the predilection of mandibular bone infection. Impairment of leukocyte function and decreased vascular supply to the hyperdense area of bone could reduce antibiotics efficacy in the infected area and precipitate chronic mandibular osteomyelitis (García et al., 2013).

### 4.1 RANKL‐dependent osteopetrosis: A clinical review

*RANKL* or *TNFSF11* was a causative gene for osteoclast‐poor osteopetrosis. It contributed to 2% of all autosomal recessive osteopetrosis in a large cohort (Palagano et al., 2018). Albeit its small percentage, determining whether the patient harbored *RANKL* variants or not is essential as hematopoietic stem cell transplantation is not effective for RANKL‐dependent osteopetrosis. Treatment for this specific genotype has shifted its focus on replacement of soluble RANKL instead.

Physiologically, RANKL exerts its function through RANKL/RANK (receptor activator of nuclear factor kappa‐B)/OPG (soluble decoy receptor osteoprotegerin) system. The pathway is involved in bone remodeling, immune regulation (osteoimmunology), and tumorigenesis (Infante et al., 2019; Walsh & Choi, 2014). When investigates its structure, TNF‐like core domain is an important domain. There is a secondary structure of 10 β‐strands that facilitate homotrimerization of RANKL and three conserved residue that were predicted to be crucial in its interaction with RANK (Lam et al., 2001). Variants fall in this domain are subjected to have a potential impact in RANKL function.

Regarding ACMG guideline for standard interpretation, the homozygous variants NM_033012.3 (*TNFSF11*): c.623T>G, p.Phe208Cys observed in these siblings is considered likely pathogenic. Due to its novel, missense change variants, potential feasible laboratory confirmation of the variants to fulfil its pathogenicity per ACMG standards and guidelines would be a functional study. There is no established gold standard for functional study of RANKL; however, multiple potential functional studies were published. A comprehensive example would be the study of an effect of variants in a rodent, as an osteoclast‐poor Tnfsf11tler/tler (toothless) mouse model is well established (Douni et al., 2012). Another functional study in literature was a study of inhibitory function of mutated RANKL in osteoclastogenesis assays (Cheng et al., 2009). This study would fit as a functional assay to test an impairment of RANK and mutated RANKL binding at their interface. In our case, determination of homotrimerization capacity would be more suggestive as a functional study as the variants was predicted to disrupt β‐strand F, a structure that involved in RANKL homotrimerization. Performing bone biopsy to demonstrate a lack of osteoclast might also be considered as it was more feasible. However, we did not further perform such a procedure as the lack of osteoclast is not pathognomonic for RANKL‐dependent osteopetrosis, and the procedure would not immediately benefit them. Further functional studies mentioned above are appreciable in light of novel specific treatment.

There were seven families of nine RANKL‐dependent osteopetrosis published from a large osteopetrosis cohort (Lo Iacono et al., 2013). Their phenotypes and molecular diagnosis were presented in comparison with our two patients in Table 1. Overall, our siblings’ phenotypes were milder than any previous reports (Lo Iacono et al., 2013; Nicholls et al., 2005; Sobacchi et al., 2007). The onset of RANKL‐dependent osteopetrosis is not limited to neonatal or infantile period as our siblings demonstrated apparent symptoms in their childhood. Hematologic involvement does not occur in all case, and serum calcium concentration may vary from hypocalcemic seizure to normal range. The common findings in all cases, including our cases, are skeletal dysmorphism (growth retardation/short stature), impairment of dentition, and compressive cranial neuropathy. Pathologic fractures were frequently found, and osteomyelitis of different bones (mandible, toe) occurred occasionally even with normal immunological studies in each patient.

Absence or impairment of RANKL causes osteopetrosis. When we looked into the mutational spectrum of this disease, there were a wide range of predicted functional variants including exon‐skipping, nonsense, frameshift and missense variants. The majority of the variants were in a functional domain which is crucial for RANKL trimerization. All variants reported were presented in homozygous state. The variants c.596T>A, p.Met199Lys occurred repeatedly in “Sikh” families, and this might be contributed by a founder effect. As for the other variants, histories of consanguinity were reported in all three families. Generally, there was no apparent genotype–phenotype correlation between *RANKL* and osteopetrosis. The observation was quite limited as phenotypes from two families, both having c.596T>A and p.Met199Lys, were not reported. Our addition of novel variants should be further observed to determine whether the variants resulted in milder phenotype than the other, or there was the work of other gene(s) found in Thai population.

The molecular diagnosis of *TNFSF11* alone excluded the patients from hematopoietic stem cell transplantation. Consensus guidelines for the management of osteopetrosis for whom hematopoietic stem cell transplantation is not the standard of care have been recently reviewed by Wu et al. (2017). Specific treatment for RANKL‐dependent osteopetrosis is a topic of interest. Previous functional study in mice model showed that administration of RANKL in a RANKL‐null mouse resulted in increased number of osteoclast and its bone resorption activity (Sobacchi et al., 2007). Multiple preclinical studies for treatment of *RANKL* osteopetrosis are ongoing, awaiting its translation to clinical trials (Cappariello et al., 2015; Menale et al., 2019). There has not yet been a study of RANKL pharmacotherapy for *RANKL* osteopetrosis patients. Our patients might be eligible for such pharmacotherapy in the future.

As for other general form of osteopetrosis, a clinical trial on interferon gamma −1b for intermediate osteopetrosis had just completed its phase 2 in April 2019, and results are pending (ACTIMMUNE in Intermediate Osteopetrosis; ClinicalTrials.gov Identifier: NCT02666768). The drug demonstrated no effectiveness in decreasing bone mass in autosomal dominant osteopetrosis (Imel et al., 2019). Our patients are appropriately followed by geneticist, endocrinologist, ophthalmologist, otolaryngologist, and dentist. As the field is currently advancing and scarcity of patients is evident, future international multicenter study would be helpful.

In conclusion, we reported the mildest phenotypic spectrum of RANKL‐dependent osteopetrosis in Thai siblings. There is no established genotype‐phenotype correlation at the moment. *RANKL* should be included in genetic evaluation across neonatal to intermediate form of osteopetrosis. There is no specific treatment up to date, but preclinical studies are on‐going awaiting its clinical translation.

## CONSENT

Written informed consent was obtained from patients and their parents for publication of this case report and any accompanying images. A scanned‐original copy of the written consent is available for review by the Editor‐in‐Chief of this journal.

## ETHICAL COMPLIANCE

The study protocol was approved by the Siriraj Institutional Review Board (Protocol No. 553/2562(EC1)). The study was conducted in accordance with the Good Clinical Practice and the Declaration of Helsinki. The patients and their parents provided informed consents prior to participation.

## CONFLICT OF INTEREST

The authors have declared that they have no conflict of interests exist.

## Data Availability

The data that support the findings of this study are available from the corresponding author upon reasonable request.
